# True functional ability of chronic stroke patients

**DOI:** 10.1186/1743-0003-10-20

**Published:** 2013-02-13

**Authors:** William J Tippett, Lisa D Alexander, Mireille N Rizkalla, Lauren E Sergio, Sandra E Black

**Affiliations:** 1 L.C. Campbell Cognitive Neurology Research Unit, Toronto, ON, Canada; 2 Sunnybrook Health Sciences Centre Research Institute, Toronto, ON, Canada; 3 Heart and Stroke Foundation Centre for Stroke Recovery, Toronto, ON, Canada; 4 School of Kinesiology and Health Science, York University, Toronto, ON, Canada; 5 Centre for Vision Research, York University, Toronto, ON, Canada; 6 Department of Medicine at Sunnybrook Health Sciences Centre & University of Toronto, Toronto, ON, Canada; 7 Department of Psychology, University of Northern British Columbia, 3333 University Way, Prince George, BC, Canada

**Keywords:** Stroke, Visuospatial, Visuomotor, Variability, Functional

## Abstract

**Background:**

There is a paucity of information regarding visuospatial (VS) and visuomotor (VM) task performance in patients with chronic right fronto-parietal lobe stroke, as the majority of knowledge to date in this realm has been gleaned from acute stroke patients. The goal of this paper is to determine how VS and VM performance in chronic stroke patients compare to the performance of healthy participants.

**Methods:**

Nine patients with stroke involving the right fronto-parietal region were evaluated against match controls on neuropsychological tests and a computerized visuomotor assessment task.

**Results:**

Initial evaluation indicated that performance between participant groups were relatively similar on all measures. However, an in-depth analysis of variability revealed observable differences between participant groups. In addition, large effect sizes were also observed supporting the theory that using only conventional examination (e.g., p-values) measures may result in miss-identifying crucial stroke-related differences.

**Conclusion:**

Through conventional evaluation methods it would appear that the chronic stroke participants had made significant functional gains relatively to a control group many years post-stroke. It was shown that the type of evaluation used is essential to identifying group differences. Thus, supplementary methods of evaluation are required to unmask the true functional ability of individuals many years post-stroke.

## Introduction

Visuospatial performance deficits and impaired visually-guided motor control are often reported in individuals with right hemispheric stroke [1,2]. Specifically, right hemispheric damage involving the fronto-parietal region is thought to affect several sub-processes of visually-guided movements such as goal-directed reaching [3,4], grasping [5,6] and pointing [7]. Right hemispheric lesions have also been associated with reductions in reaction time, in the planning of visually-guided movements, and in degraded hand kinematics, specifically when using the contralesional limb [8,9].

Among the few studies conducted on chronic stroke patients, who may be more likely to exhibit stable performance characteristics, investigators have probed either visuospatial or visuomotor performance [10-12], but not both. Obtaining information on both types of task performance in chronic right fronto-parietal lobe stroke patients may provide insight into the effects of damage to potentially overlapping functional networks required for these tasks.

The research provided within this document suggests that characterizing life-long VS/VM deficits can be challenging and requires sensitive tools in order to provide an accurate representation of one’s cognitive and cognitive-motor integration abilities after considerable time post stroke.

Thus, in this investigation, VS and VM performance in a group of chronic stroke patients with substantial right fronto-parietal lobe damage was compared to an age-matched control population to ascertain how performance in patients who were many years post-ictus compared to that of healthy participants. Therefore, it was hypothesized that compared healthy individuals stroke patients would display significant difficulty on standard VS & VM tests and diminished visually-guided motor performance, particularly with the presence of damage to right fronto-parietal lobe. The primary question to be examined is: Do current standard testing measures provide a true characterization of chronic stroke patient’s ability as it relates it visually guided motor control?

## Methods

### Participant characteristics

All available patients (6 men and 3 women mean age 56.7 ± 13.2 years) who exhibited stroke involving the right fronto-parietal lobes, were recruited from a pool of community-dwelling chronic stroke patients being followed through the Cognitive Neurology Clinic and the Heart and Stroke Foundation Centre for Stroke Recovery at Sunnybrook Health Sciences Centre (SHSC), a University of Toronto academic healthcare institution. Patient demographic and clinical information is listed in Table 1. Patients were on average 8.9 years (range 3.3-19 years) post-stroke, initially presented with a symptoms suggesting right hemisphere damage, due to right middle cerebral artery infarction. In certain cases, there was ablation of nearly the entire MCA territory, and a few patients also had significant damage in the anterior cerebral artery territory. All patient participants underwent standard rehabilitation intervention (e.g., occupational and physical therapy) in the subacute phase of their stroke.

**Table 1:** Patient demographic data and neuropsychological scores

**Patient ID**	**1**	**2**	**3**	**4**	**5**	**6**	**7**	**8**	**9**
**Age**	53	59	51	42	57	48	69	20	45
**Sex**	M	M	M	M	M	M	F	F	F
**YOE**	14	8	17	11	15	13	11	12	13
**Total Lesion Volume (cm**^**3**^**)**	127	23	70	49	190	99	51	232	333
**MMSE**	30	26	29	29	30	27	27	29	29
**NIHSS Neglect Scores**	0	0	0	1 (tactile)	0	0	0	1 (tactile)	0
**Mean IT (sec)**	0.73	1.12	0.74	0.85	0.89	1.43	1.71*	0.74	1.27
**Mean MT (sec)**	0.47	0.52	0.45	0.82*	0.83*	0.42	0.77	0.38	0.40
**Clock Drawing**	10	10	10	10	n/a	n/a	10	10	10
**Trails A (sec)**	25	67*	n/a	31	37	25	66*	7	29
**Trails B (sec)**	42	188*	n/a	57	141	80	240*	13	75
**Benton**	n/a	n/a	26	n/a	23	24	20	13	16
**Rey-O (copy)**	35	30	36	35	n/a	24.5*	29	19.5*	29.5
**Rey-O (IR)**	25	10.5	28	22	n/a	13.5	9	7.5	10.5
**Rey-O (DR)**	25	13	26.5	23	n/a	8	10	8.5	13.5
**Sup parietal >10%**					**Y**				Y
**Inf parietal >10%**	Y	**Y**			**Y**	**Y**		**Y**	Y
**Supramarg >10%**		**Y**		**Y**	**Y**	**Y**		**Y**	Y
**Mid Frontal >10%**	Y								
**Inf frontal >10%**	Y		Y	**Y**	**Y**	**Y**	**Y**	**Y**	
**BG >10%**			Y	**Y**	**Y**	**Y**	**Y**		Y

Demographic data and neuropsychological scores for each patient are shown above. ‘Age’ indicates age at time of stroke, years of education (YOE),Mini Mental State Exam (MMSE), initiation time (IT), movement time (MT), ‘Clock Drawing’ indicates total Clock Drawing Assessment scores, Benton Line Orientation scores (Benton), Rey-Osterrieth complex figure test (Rey-O copy), ‘Rey-O IR’ indicates immediate recall Rey-Osterrieth score, ‘Rey-O DR’ indicates delayed recall Rey-Osterrieth score, and ‘n/a’ indicates data not available. Values for patients that exhibited impairment on visuospatial tests are bolded with an asterisk. Patients were considered as impaired on visuomotor testing if MT or IT was greater than 2 standard deviations when converted to z-scores.

Control participants were age, education and sex matched to the patient sample for the paper-based Neuropsychological VS/VM assessments, which included 4 men and 5 women with a mean age 60.7 ± 3.9 years. One-way analysis of variance conducted between participants groups using corrected values indicated no significant difference between sample populations on age, education and sex variables. In addition, a second sub-set of 11 normal control participants completed the computer-based visuomotor procedure (7 men and 4 women, mean age 53.6 ± 14.7 years). Likewise, one-way analysis of variance on demographic characteristics indicated no significant difference on age, education and sex variables. All research participants were right handed and demonstrated understanding of tasks instructions.

All control participants were community dwelling healthy volunteers with no significant medical or neurological history. All participants provided written informed consent to participate in this research study, which was approved by the SHSC Research Ethics Board.

### Neuropsychological visuospatial testing

Seven neuropsychological tests were utilized to assess VS/VM function. The Benton Judgement of Line Orientation (BLO) [13], was included as it has been shown to correlate with right parietal damage [13,14] and is one of few noted “pure” visuospatial measures. Research has noted that the right parietal regions have also been shown to be active during BLO task performance using functional neuroimaging [15].

Further, recent evidence from functional imaging and lesion studies suggests that both right frontal and parietal regions may be involved in VM task performance and in VS tests that have an “executive type” components involved in task completion, thus tasks such as Trails A and B and the Rey-Osterrieth Complex Figure test were included in this examination [16,17]. Accordingly, the Rey-Osterrieth complex figure test (copy, immediate recall, delayed recall) is noted to probe the parietal-frontal network [18] which is related to both spatial analysis and frontal executive processes, [19,20] as well as, to regions mediating visual memory [21-24]. The Trails A task, a measure of psychomotor speed, and the Trails B, a measure of attentional and executive processes implicating the parietal-frontal “networks” [25,26] were also administered. Clock Drawing was included as a measure to assess visuoconstruction ability [27]. Acute estimate of neglect behaviour was derived from the extinction and inattention (neglect) sub-domain of the National Institutes of Health Stroke Scale (NIHSS), where a score of 0 indicates no abnormality, 1 indicates the presence of mild visual, tactile or auditory extinction or neglect, and 2 indicates profound extinction or neglect (measurement taken at first assessment after post-stroke episode). Lastly, all participants completed the Mini-Mental Status Exam (MMSE), which examines orientation to time and place, attention and calculation, recall, language, and visual construction [28].

### Computerized visuomotor assessment

**Figure 1:**
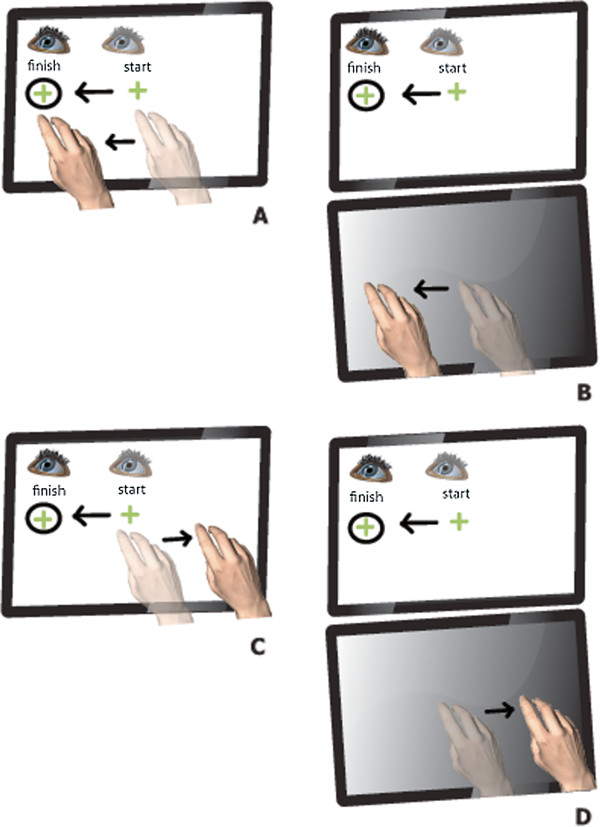
**All conditions randomly display targets in 0°, 90°, 180° or 270° positions.** Touch screen location is altered in conditions **B** &**D** and visual feedback of cursor position is also altered in conditions **C** &**D**. **A**) Displays the direct 1–1 condition where finger position is cursor position. **B**) Displays the touch screen placed horizontally in front of the vertical monitor, participants move their finger on horizontal touch screen to observe movements on the vertical screen. **C**) Touch screens is placed over vertical monitor as in **A**, however on screen cursor moves 180° from that of finger movement. **D**) Touch screen placed again horizontally as in **B** and cursor moves as described in **C**, 180° away from finger position. Figure 1 is reprinted with permission from Elsevier Ltd.

Participants completed a computer-based visuomotor task (CbVM) (adpated from Tippett & Sergio 2006 [29]), designed to examine visually-guided motor performance and postulated to probe the right parietal function involved in point-to-point hand movements. The task is thought to engage a parietal-frontal “network” involved in choosing targets of interest and transforming the target information into an appropriate movement goal [30]. Participants were required to slide their finger over a touch-sensitive screen in order to displace a cursor viewed on a computer monitor (arrow) to specific target locations. Specifically, participants performed the task under one of two cognitive conditions using one of two visuospatial mappings between target viewing and hand motion. Four 25 mm diameter targets were presented 80 mm from a central target along the four cardinal axes on the screen at 0°, 90°, 180° and 270°, first in a direct one-to-one procedure (e.g., finger moves left and cursor moves left) and second, in a rotated “nonstandard” visual feedback condition (e.g., finger moves left and cursor moves right). In addition, two separate spatial locations were used for the touch screen: first the touch screen was placed directly on the monitor, and second, the touch screen was placed horizontally on the table in front of the monitor (Figure 1). Each of the 4 conditions involved 20 trials and took approximately 15–20 minutes to complete. During task performance, movement times (MT) and initiation times (IT) were collected and summed to create a total reaction time (RT) score. IT was calculated by scoring initial onset of finger movement (i.e. when the participant moved from centre target position), and MT was calculated by the total time required to move to the target position after the target was presented. For further descriptions of the task, see [29].

### Image acquisition and analysis

Anatomical imaging was performed on a research MRI scanner (1.5 T, GE Medical Systems, software version LX 8.2.5, NV/i hardware platform). A standard three-dimensional fast spoiled gradient-echo anatomical imaging sequence was used to obtain T1-weighted images (Repetition time = 12.4 ms; Echo time = 5.4 ms; Flip Angle, θ = 35°; Acquisition Matrix = 256x192; Slices = 124; Slice Thickness = 1.4 mm; Field of View = 22x16 cm). Image alignment, lesion tracing and lesion volume calculations were completed by a trained image analyst using ANALYZE 6.0 software (Biomedical Imaging Resource, Mayo Foundation). Tracing was performed blind to all clinical data except the side of hemiparesis. Lesions were visually identified as having low signal intensity in relation to homologous contralateral tissue. Both the black core (CSF intensity) and dark peri-infarct regions were included in the tracing. Incidental lacunar infarcts were also included in lesion tracings. The tracings were co-registered to the Montreal Neurological Institute (MNI) brain template using 16 non-linear transformations and cost function masking [31] and region of interest (ROI) images were generated using MRIcro software (http://www.mccauslandcenter.sc.edu/mricro/mricro/mricro.html). ROI images were then overlapped to indicate the frequency of damage for each voxel. The Talairach coordinates [32] acquired in MRIcro were used to identify relevant anatomical structures implicated in the ROI analyses.

### Statistical analysis

An analysis of variance (ANOVA) was used to examine the main effects of VS/VM measures between and within participants groups (critical value p >0.05). It is important to note that for the Benton line orientation test data was retrieved for 6 of 9 of patients (2 patients were not within our testing time constraints and 1 patient failed to pass screening). To evaluate CbVM performance, we conducted a MANOVA (multivariate) analysis to examine the main effects of performance (e.g., MT and IT) between participant groups. VM performance was considered as abnormal when z-scores, determined using the SD and mean scores of normal controls, for initiation times or movement times were > 2. Data were analyzed using SPSS for Windows (version 15; SPSS Inc., Chicago, IL).

Effect size calculations were also conducted to describe the degree to which experimental differences were observed between our two populations. Effect size calculations are particularly useful when faced with low statistical power, as they have the ability to demonstrate the overall magnitude of relationships. Therefore, as recommended by Zakzanis (2001), Cohen’s d was utilized as it is identified to be the most appropriate for use with patient participants engaging in neuropsychological evaluations [33]. The use of effect size also provides a way to measure significance, particularly in this context, given the paucity of precedent analysis examining VS and VM task performance in chronic stroke patient.

### Performance consistency

It is commonly observed that brain injured individuals tend to exhibit greater performance diversity compared to normal controls [34-37]. Inter-individual variation can be thought of as inconsistency in ability of individuals in an identified group, whereas intra-individual variability is temporary alterations in performance by an individual engaged in a one-time measure of their performance [38]. Both forms of variability (inter and intra) can be viewed as `noise' [38]. Performance variability and its inverse, performance consistency, may be a source of considerable information, not to be overlooked [39-42]; specifically, past research on stroke patients has noted that reaching for a target can be met with overall success when evaluated against controls, however, the mechanisms used (e.g., degrees of freedom) to achieve this goal can be quite different[43,44]; and thus studying variability as the dependent measure of interest can be informative [45,46]. For this study, the measure of inter-individual performance variability was calculated as the mean of the standard deviations for IT, MT on the CbVM, with variability of the neuropsychological tests further characterized between stroke patients and controls. This analysis examined whether the mean of all the individual variances (standard deviation squared for each group) was equivalent between the two groups. The Coefficient of Variation (ratio of group standard deviation to the group mean) was calculated as an additional measure of variability [38]. The measure of intra-individual variability was calculated as the standard deviation for each individual within the four conditions on CbVM using IT and MT values.

## Results

### Neuroimaging

**Figure 2:**
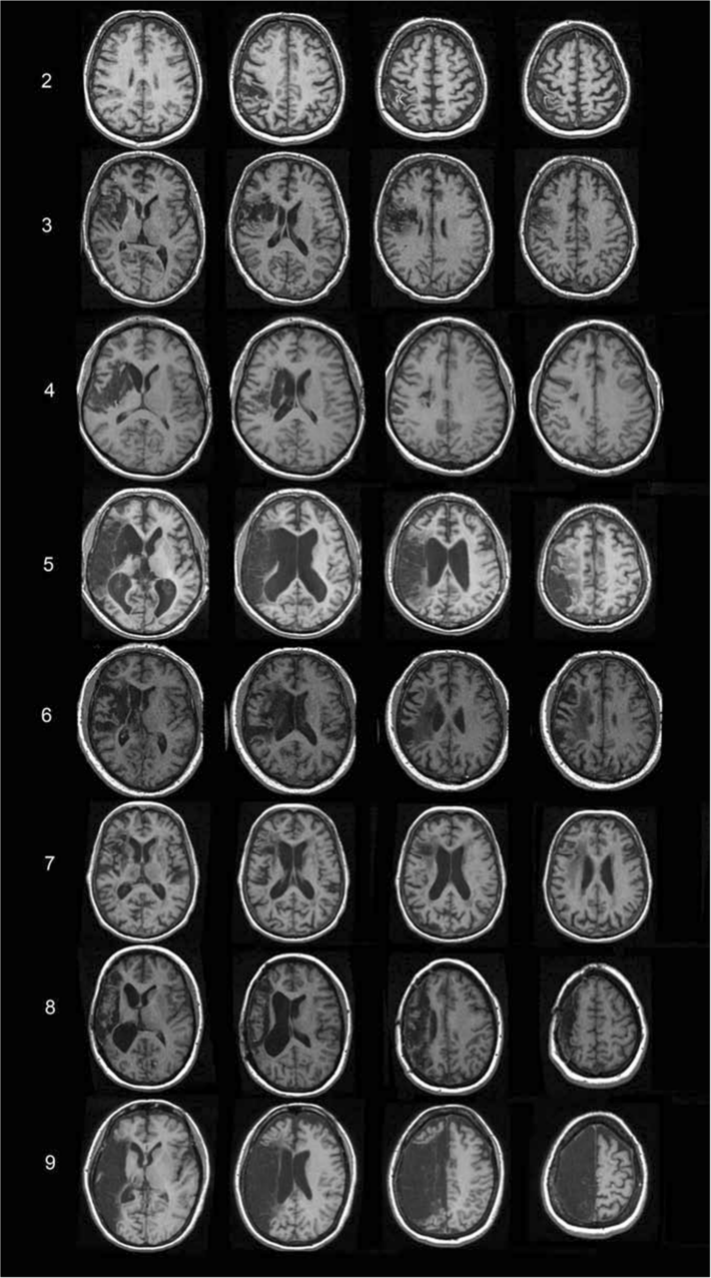
**Four representative slices of stroke lesion territory are shown for 8 of 9 stroke patients.** The numbers along the left side of Figure 2 correspond to the ID numbers in Tables 1 and 2. Although processed lesion data was available for patient #1, the raw MR images were not locatable.

**Figure 3:**
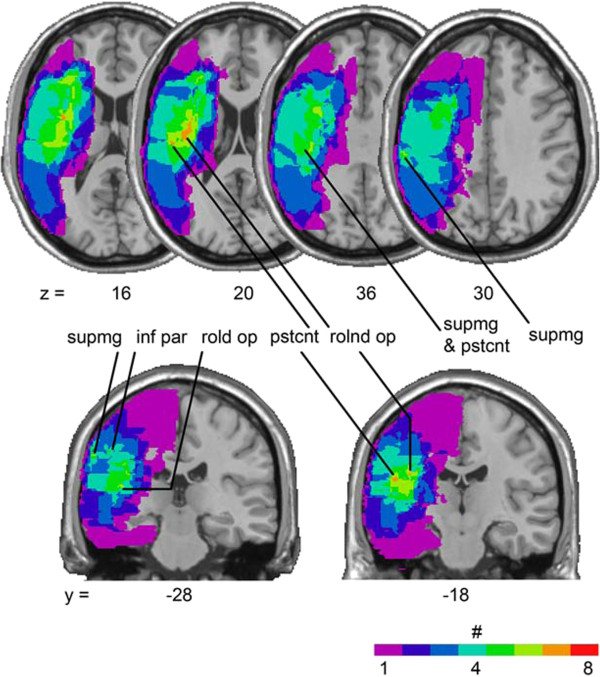
**Overlay of lesions for 8 patients with stroke involving the right parietal lobe.** Voxels damaged in one patient are shown in purple and shades toward the red end of spectrum denote voxels where larger numbers of patients were lesioned, as indicated in the key. ‘Supmg’ indicates supramarginal gyrus, ‘inf par’ indicates inferior parietal lobule, ‘pstcnt’ indicates postcentral gyrus, ‘rolnd op’ indicates rolandic operculum.

All patients experienced ischemic stroke involving the right hemisphere, and the mean time for MRI was 42.4 months (± 45.1 months, range 2–122 months) post-stroke. Figure 2 displays four representative MRI slices for each patient showing the lesioned brain regions, and Table 2 shows the percentages of tissue that was lesioned in various anatomical regions. The mean patient lesion volume was 124.31 cm^3^ (± 106.21, range 41.0-324.9 cm^3^). The overlay of patients’ ROIs indicated that lesions exhibited maximal overlap in regions of the supramarginal gyrus, inferior parietal lobule, postcentral gyrus and rolandic operculum (Figure 3).

**Table 2:** Percentages of lesioned tissue in frequently damaged anatomical regions

**Anatomical Region:**	**Patient 1**	**Patient 2**	**Patient 3**	**Patient 4**	**Patient 5**	**Patient 6**	**Patient 7**	**Patient 8**	**Patient 9**
Postcentral	42.1%	16.5%	29.8%	3.9%	49.6%	9.0%	0.7%	56.8%	78.3%
Superior Parietal	0.0%	6.3%	0.0%	0.0%	12.5%	0.0%	0.0%	5.3%	59.1%
Inferior Parietal	24.5%	43.3%	0.7%	2.0%	83.5%	17.8%	0.0%	78.4%	75.3%
Supramarginal Gyrus	0.0%	14.8%	2.4%	27.1%	84.0%	63.5%	0.0%	79.1%	69.8%
Angular Gyrus	0.0%	0.0%	0.0%	0.0%	76.0%	6.5%	0.0%	66.8%	72.9%
Precuneus	0.0%	0.2%	0.0%	0.0%	0.4%	0.0%	0.0%	0.0%	46.1%
Paracentral Lobule	0.0%	0.0%	0.0%	0.0%	0.0%	0.0%	0.0%	0.0%	83.8%
Caudate	0.0%	0.0%	25.1%	34.0%	0.0%	24.1%	0.7%	0.0%	2.4%
Putamen	0.3%	0.0%	69.9%	90.3%	52.2%	65.6%	46.6%	1.5%	33.4%

Patients’ lesions frequently involved the anatomical regions listed above. The percentage of lesioned tissue for each of these individual regions is indicated, which was determined by dividing the number of lesioned voxels in each region by the total volume of the region.

### Neuropsychology evaluation

One way analysis of variance (ANOVA) between groups for visuospatial measures showed no significant differences for the Rey-Osterreith figure copy (F_1,16_ = 2.56, p >0.05), Rey immediate recall (F_1,15_ = 1.15, p >0.05), Rey delayed recall (F_1,15_ = 1.34, p >0.05) and MMSE (F_1,16_ = 0.0, p >0.05). Likewise, there were no significant main effects for group on the Trails A (F_1,14_ = 0.33, p >0.05), Trails B (F_1,13_ = 1.72, p >0.05), or Clock Drawing (F_1,14_ = 1.75, p >0.05). Scores on the BLO were significantly different between stroke patients and the control group (F_1,12_ = 12.24, p <0.005). There were no within group effects observed (p >0.05). Two of nine patients exhibited tactile extinction, each scoring 1 point on this section of the NIHSS. No patients were found to have visual extinction. On 9 separate occasions, patients fell below normal limits (NL) values and on 10 separate occasions, patients either declined to perform certain neuropsychology tasks or did not pass the initial screening procedures.

### Computerized visuomotor procedure

Across all four CbVM conditions, the mean reaction time for the stroke patients was .56 ± .50, .80 ± .40, .80 ± .21 and 1.1 ± .52 seconds, respectively. The mean reaction time for the control participants on all four visuomotor measures were as follows: .63 ± 5.0, .78 ± .61, .85 ± .46 and .93 ± .53. Multivariate analysis (MANCOVA) revealed no significant main effect between groups based on average reaction time values for each participant (F_1,18_ = p >0.05).

### Individual analysis

Despite what appears as a relative comparable performance in this stroke population sample, further investigation (Table 1) showed that 4 patients with at least 1 abnormal neuropsychological test (Trails or Rey-copy, patients 2, 6, 7, 8), and 3 patients had an abnormal CbVM scores (patients 4, 5, 7).

**Figure 4:**
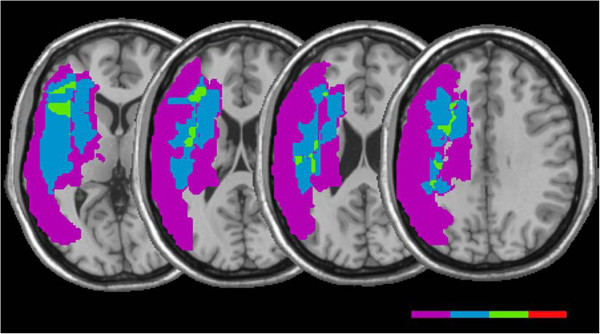
**Overlay of patients with 1 abnormal VS test score.** Purple indicates area of lesion involvement for 1 patient, blue for 2 patients, green 3 patients, red 4 patients. Commonly involved regions included the anterior-inferior frontal, mid frontal and medial supramarginal regions.

**Figure 5:**
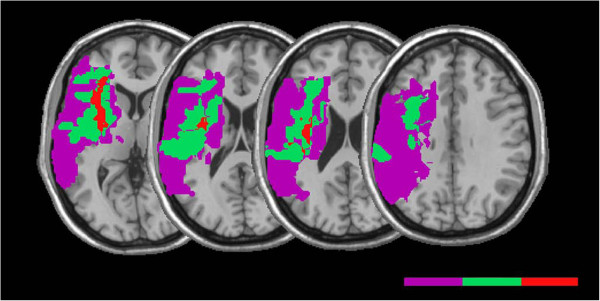
**Overlay of patients with 1 abnormal VM test.** Purple indicates area of lesion involvement for 1 patient, green 2 patients, red 3 patients. Commonly involved regions included the inferior frontal (red), basal ganglia (red) and supramarginal regions (red).

A lesion overlay of patients with an abnormal neuropsychological test showed that the anterior-inferior frontal, mid frontal, centrum semiovale and supramarginal regions was commonly involved in this group (Figure 4). Commonly lesioned regions for patients with an abnormal VM score included the anterior-inferior frontal, basal ganglia, centrum semiovale, corona radiata and supramarginal regions (Figure 5). A lesion overlay of all patients with either an abnormal CbVM or neuropsychological score showed maximal overlap in the inferior frontal, basal ganglia, centrum semiovale, and supramarginal regions, which suggests that the CbVM and neuropsychological tasks may rely on common structural regions for successful task completion. CbVM or neuropsychological deficits were noted in patients with lesions involving one or all of these regions, See Table 2 (all regions were not necessarily lesioned in patients that exhibited performance deficits).

### Effect size

In an effort to understand the magnitude of relationships for each measure, effect size values were calculated. The results demonstrate that half of the testing measures used in this experiment did in fact reach a large effect size value (range .76-1.87), which can be observed on both neuropsychological VS/VM tasks and on the CbVM measure. The effect size results presented in Table 3 show that individuals post sub-acute phase of a stroke episode still display significant impairments on a number of the neuropsychological tasks. In addition, to this reduced ability significant differences were also observed on the CbVM as noted by the t-test results in Table 4.

**Table 3:** Representative effect size values using Cohen’s d

**Test measures**	**Cohen’s d**
IT 1	.25
IT 2	.04
IT3	.41
IT4	-.11
MT1	-.48
MT2	.46
**MT3**	-**.76**
**MT4**	**-.86**
**Rey Copy**	**1.2**
**Rey Imm. Recall**	**.78**
**Rey delay**	**-.96**
**MMSE**	**-.91**
Trails A	.40
Trails B	.63
**Clock Draw**	**.96**
**Benton Line O**	**1.9**

Table 3 displays the effect size values (Cohen’s d) for each of the testing measures. The bolded values indicate less than 50% overlap between groups.

**Table 4:** **Independent samples *t*-test, significance levels and 95**% **confidence intervals**

				**95% C.I. for Odds ratio**	
**Condition**	***T***	***Df***	**p**	**Lower**	**Upper**
IT1	2.53	17	.022*	21.56	239.14
IT2	4.33	17	.000*	248.60	722.24
IT3	2.44	17	.026*	17.03	235.67
IT4	2.65	17	.017*	65.75	581.48
MT1	2.69	17	.016*	11.98	99.27
MT2	2.43	17	.026*	13.65	193.85
MT3	2.67	17	.016*	33.22	284.05
MT4	3.75	17	.002*	76.37	272.91

* *p* < .05.

Table 4 displays the analysis of group variances for all IT and MT conditions on the CbVM.

### Inter-individual variability

**Figure 6:**
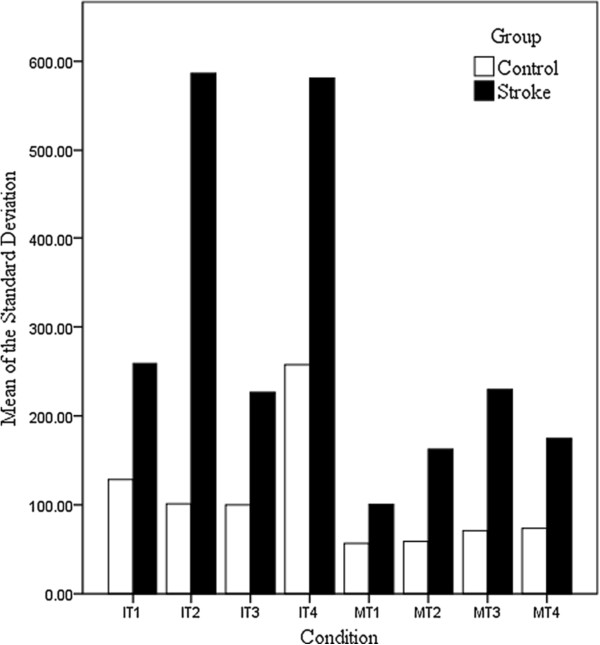
**Comparing the mean of the standard deviation between groups on IT and MT.** The stroke group (black) shows greater variability than the control group (white) for all conditions.

**Figure 7:**
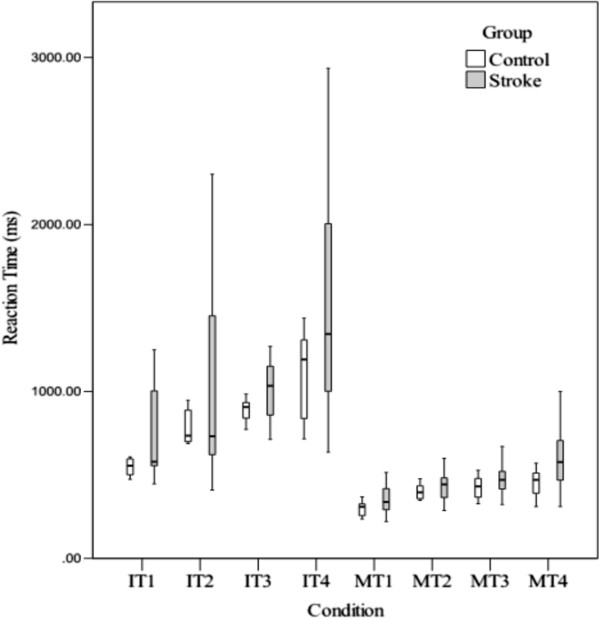
**Comparing spread of distribution around the mean IT and MT within a group.** The stroke group has larger spread than the controls for all the conditions.

In the analysis of group variances, the stroke participants show greater performance fluctuation for all IT and MT conditions (See Table 4). Figure 6 shows the striking difference in variability between the groups for each condition. Figure 7 shows the spread of variance around the mean RT within a group. Notably, the stroke participants always had a larger spread than the controls. The stroke group was also significantly different from the controls in variability for the Rey, Trails A and Trails B. However, there was no significant difference in variability between the groups for the BLO. Table 5 shows the results of inter-individual variability for the BLO, Rey, Trails A and Trails B.

**Table 5:** Inter-individual variability for the BLO, REY, Trails A and Trails B

				**95% C.I. for Odds ratio**	
**Test**	***T***	***df***	**p**	**Lower**	**Upper**
REY	2.18	14	.047	0.11	12.40
BLO	0.43	13	.671	2.35	3.54
Trails A	2.43	15	.028	−153.04	−9.92
Trails B	2.9	15	.010	−78.26	−12.35

The stroke group was significantly different from the controls in variability for the REY, Trails A and Trails B. There was no significant difference in variability between the groups for the BLO.

In addition as shown in the CbVM results, the average IT and MT coefficient of variation for the patient and control groups indicated significant differences in variability, with the patient group having greater coefficient of variation for IT (0.45 vs. 0.22) and MT (0.38 vs. 0.24) than controls. Table 6 has a breakdown for each condition, which also demonstrated that all patients’ responses (as displayed via means and standard deviations) were above that of the control group.

**Table 6:** Reaction time characteristics and coefficient of variability for the two groups

	**Group**					
**Condition**	**Stroke**			**Control**		
	**Mean**	**Std. Dev.**	**Coefficient**	**Mean**	**Std. Dev.**	**Coefficient**
IT1	745.55	290.94	0.40	628.66	176.09	0.28
IT2	1101.93	715.90	0.65	791.51	111.75	0.14
IT3	1009.56	243.64	0.25	1008.00	195.82	0.19
IT4	1497.08	711.53	0.48	1098.72	291.98	0.27
MT1	369.81	88.71	0.24	328.80	78.18	0.23
MT2	501.12	206.15	0.41	405.30	135.55	0.33
MT3	598.92	303.75	0.51	430.11	88.84	0.21
MT4	599.02	218.51	0.36	453.49	86.42	0.19

Stroke group shows greater coefficient of variability than control group for all IT and MT conditions.

### Intra-individual variance

**Figure 8:**
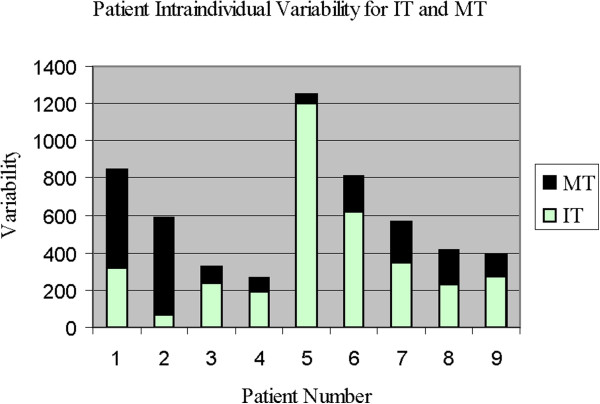
Intra-individual variability for IT and MT showing pronounced IT variability for frontal and frontal-parietal subjects (3–9 ) and pronounced or MT variability for parietal subjects (1, 2).

We undertook a comparison of IT and MT intra-individual variability for each stroke patient according to lesion location (parietal vs. frontal vs. both frontal-parietal, Table 7). We observed that individuals with greater frontal regional damage demonstrated consistently higher variability on IT than MT, and vice-versa for individuals with greater parietal regional damage. Moreover, there was an overall strong correlation between injury severity and the measure of variability (r = .69, p < .05). Individuals with damage to both parietal and frontal areas also tended to exhibit higher variability on IT measures. Figure 8 displays the intra-individual variability for IT and MT for each participant. Importantly, one can readily observe a pronounced variability in one of the conditions (IT or MT) depending on the site of lesion (frontal or parietal, respectively).

**Table 7:** Intraindividual variability for each stroke patient according to lesion location

		**IT**		**MT**	
**Lesion**	**Subject**	**Mean**	**SD**	**Mean**	**SD**
Frontal	3	1268.90	236.37	396.58	88.94
	4	738.93	190.00	380.28	76.04
	7	827.89	344.23	888.73	222.55
Parietal	1	765.46	318.09	1707.61	530.91
	2	422.07	65.27	1434.47	523.24
Frontal-Parietal	5	1162.71	1200.10	425.89	50.40
	6	1123.88	615.72	520.16	193.74
	8	727.16	226.14	466.27	187.01
	9	740.90	276.31	450.36	114.80

Patients with frontal damage demonstrate higher variability on IT whereas patients with parietal damage demonstrate higher variability on MT. Patients with damage to both parietal and frontal areas also exhibit higher variability on IT measures. Patient 7 (frontal case) is an exception to the pattern with MT higher than IT. However, MT of patient 7 is still much lower than all other parietal lesion cases.

## Discussion

Previous investigations have reported that VS/VM deficits occurring in stroke patients with right parietal lobe injury often persist into the chronic stage of stroke [47,48] and that patient’s with right parietal lesions often exhibit deficits on VS/VM tasks similar to those used in the present study. For example, Schaefer and colleagues (2007) found that 5 right hemisphere-damaged chronic stroke patients (average of 7.8 years post-stroke), the majority of whom exhibited some parietal lobe injury, demonstrated impaired final positioning and prolonged movement duration during targeted right arm movements [2]. Likewise, Hermsdorfer et al. (1999) reported that 9 chronic stroke patients (average of 8 months post-stroke) whose lesions encompassed the parietal lobe showed prolonged reaction time and degraded kinematics during prehensile movements with the ipsilesional upper extremity [8]. Also, studies have demonstrated notable right parietal cortex activation in control subjects performing visuospatial tasks using near-infrared spectroscopy and fMRI [15], as well as reported performance deficits in stroke patients with right parietal lobe lesions [49].

In this study we wanted to understand if standard testing measures could provide a true characterization of chronic stroke patient’s ability as it relates “normal” visually guided motor control.

We hypothesized that our sample of chronic stroke patients with lesions involving the right fronto-parietal lobe would display marked VS/VM deficits on both conventional and novel testing procedures (e.g., CbVM) as compared to a healthy control group. Using traditional measures only, (e.g., p values) one might argue that the results were contrary to our expectations. However, though statistically a non-significant result is observed on some tasks, there were several occasions where patients fell either below normal limits, declined to perform neuropsychological tasks, or failed to pass screening, all signs lending support that patients do exhibit signs of VS/VM difficulty. As well, as shown through Cohen’s d results it’s noted that half the procedures utilized, had large effect sizes. Hence these results to a certain extent (e.g., depending on method of analysis), were able to readily distinguish between our population samples, which is remarkable for an additional reason, in that, these were community-dwelling patients participating in research and thus likely experiencing better health than counterparts at a similar stage.

Given that there are limited studies on VS/VM ability in the chronic phase of stroke, the use of effect size enables a smooth comparison to studies examining stroke patients in the acute phase or those having deficits in different cognitive domains. Here, the link between type of lesions, reaction time, and inter-individual variability was explored. Our results suggest that there may be a connection between parietal lesions and MT variability, and frontal lesions and IT variability. As shown an overall strong correlation between injury severity and the measure of variability could be observed. Interestingly, individuals with both frontal and parietal lesions appear to be more susceptible to variability in IT. Thus, it could be postulated that damage to the frontal areas results in chaotic or limited input to the parietal regions which in turn not only causes increases IT variability but also effects MT performance.

For many years the literature has documented that brain damage causes increased variability in performance [34,36,37,43,44]. The findings of this study lend support to the idea that a unique profile of abnormal variability is created by damage to either the parietal or frontal lobes. In addition, these findings support the idea that structures involved in normal VS and VM performance may operate as a network, whereby injury involving disruption of even one node of the interconnected structures may deleteriously affect task performance.

The increase in variability among the stroke group on the REY, Trails A and Trails B clarifies why these neuropsychological test were unable to expose significant mean performance differences. Evidently, the presence of variability creates “noise” that may eliminate any significant finding. The statistical noise may in fact be related to biological noise imparted into the VS/VM control system by damage to the brain, and as such makes the noise level itself the variable of interest. Analysis of neuropsychological tests requires consideration of performance variability on the tests and the lack of performance consistency demonstrated by the stroke group calls into question the accuracy of diagnosis without an examination of variability. Performance fluctuation may result in a person being classified as abnormal one day and normal the next [50]. For this reason, it is important for clinicians to have an assessment tool that could assist in monitoring performance variability. The CbVM tool used in the present study was successful in accomplishing this. Furthermore, the use of this tool may also be able to shed light on whether performance variability in brain injured populations improves with time or how this may dissociate from general slowness. Fluctuations in performance of a task may underlie some of the difficulties (such as fatigue) that are commonly reported by patients with stroke.

### Limitations

It could be argued that with a larger group size we could uncover more deficits that may be subtle and thus more difficult to identify in a smaller sample. Though this a common assumption made by many researchers, we would argue that statistically a nonsignificant result which is observed on several of the procedures examined within, does not signify that the “population effect is in fact zero; it means only that a population effect of zero cannot be ruled out” (O’Keefe 2007 p.296) ^46^. The number of available participants was limited in this investigation (due to the pool of participants we were able to draw from) and as such, we have provided the effect size values to augment standard p value reporting [33] in addition to an in-depth analysis of participant variability. We believe this provides sufficient support to distinguish sample populations, as well as, demonstrating the importance of these methods of analysis, especially if faced with sporadic patient presentation, particularly with singular patient evaluations.

## Conclusion

We make two principle observations. First, subtle performance impairments post-stroke can be quantified using a visuomotor integration assessment. Second, analyzing performance data through a quantification of reaction time variability and effect size, rather than the more standard main-effects analyses on mean reaction time values, can identify subtle yet important group differences. Together, these techniques allow one to understand the present ability of patients many years after their stroke episode to determine if performance is functionally similar to a healthy individual.

The study of variability (or its inverse, consistency) is imperative when examining the abilities of chronic patients with frontal or parietal cortex damage, since success in real-life tasks not only depends on average performance, but on predictability and consistency of performance. The results of this study demonstrate the importance of developing new methods in examining brain injured populations in regards to patient's lesion location (i.e. frontal or parietal, etc.), and assessing the specific form of variability through appropriate assessments. Future work clarifying the relationship between specific lesion sites and specific basic processes of variability could lead to more specific rehabilitation procedures and will provide a performance guideline for rehabilitation assessment.

## Abbreviations

VS: Visuospatial; VM: Visuomotor; ROI: Region of interest; BLO: Benton Judgement of Line Orientation; NIHSS: National Institutes of Health Stroke Scale; MMSE: Mini-Mental Status Exam; MT: Movement times; IT: Initiation times; RT: Reaction time; CbVM: Computer-based visuomotor task.

## Competing interests

The authors declare that they have no competing interests influencing the information provide in this document as outlined by BioMed Central Publishing Group.

## Authors’ contributions

WJT contributed to the project concept and design, data collection, data analysis, test conduction participant recruitment and writing. LA contributed to data analysis, data interpretation and writing. MR contributed in the data analysis, data interpretation and writing. LS contributed to project concept and revising manuscript. SEB contributed to participant recruitment, interpreting results, writing and revising manuscript. All authors have read and approved the final manuscript.
